# *Mycobacterium tuberculosis* Lineage Distribution Using Whole-Genome Sequencing and Bedaquiline, Clofazimine, and Linezolid Phenotypic Profiles among Rifampicin-Resistant Isolates from West Java, Indonesia

**DOI:** 10.1155/2024/2037961

**Published:** 2024-03-04

**Authors:** Andriansjah Rukmana, Cynthia Gozali, Linda Erlina

**Affiliations:** ^1^Department of Microbiology, Faculty of Medicine, Universitas Indonesia, Jakarta 10320, Indonesia; ^2^Master Programme of Biomedical Sciences, Faculty of Medicine, Universitas Indonesia, Jakarta 10430, Indonesia; ^3^Department of Medical Chemistry, Faculty of Medicine, Universitas Indonesia, Jakarta 10430, Indonesia

## Abstract

Tuberculosis (TB) is caused by *Mycobacterium tuberculosis* infection. Indonesia is ranked second in the world for TB cases. New anti-TB drugs from groups A and B, such as bedaquiline, clofazimine, and linezolid, have been shown to be effective in curing drug resistance in TB patients, and Indonesia is already using these drugs to treat patients. However, studies comparing the TB strain types with anti-TB resistance profiles are still relevant to understanding the prevalent strains in the country and their phenotypic characteristics. This study aimed to determine the association between the TB lineage distribution using whole-genome sequencing and bedaquiline, clofazimine, and linezolid phenotypic profile resistance among *M. tuberculosis*rifampicin-resistant isolates from West Java. *M. tuberculosis* isolates stock of the Department of Microbiology, Faculty of Medicine, Universitas Indonesia, was tested against bedaquiline, clofazimine, and linezolid using a mycobacteria growth indicator tube liquid culture. All isolates were tested for *M. tuberculosis* and rifampicin resistance using Xpert MTB/RIF. The DNA genome of *M. tuberculosis* was freshly extracted from a Löwenstein–Jensen medium culture and then sequenced. The isolates showed phenotypically resistance to bedaquiline, clofazimine, and linezolid at 5%, 0%, and 0%, respectively. We identified gene mutations on phenotypically bedaquiline-resistant strains (2/3), and other mutations also found in phenotypically drug-sensitive strains. Mykrobe analysis showed that most (88.33%) of the isolates could be classified as rifampicin-resistant TB. Using Mykrobe and TB-Profiler to determine the lineage distribution, the isolates were found to belong to lineage 4 (Euro-American; 48.33%), lineage 2 (East Asian/Beijing; 46.67%), and lineage 1 (Indo-Oceanic; 5%). This work underlines the requirement to increase the representation of genotype-phenotype TB data while also highlighting the importance and efficacy of WGS in predicting medication resistance and inferring disease transmission.

## 1. Introduction

Tuberculosis (TB) is one of the leading causes of death worldwide after HIV/AIDS in infectious diseases, and Indonesia is ranked second globally in terms of TB cases [1]. TB is caused by *Mycobacterium tuberculosis* infection, especially in the lungs [1, 2]. About one-third of the world's population is infected with *M. tuberculosis*, and infection with this bacterium contributes to the deaths of about two million people each year, which makes TB the eighth most common cause of death and the second leading cause of death from infectious agents. This number will increase with the emergence of drug-resistant strains of *M. tuberculosis* [3]. Indonesia is one of 10 countries attempting to prevent the incidence of rifampicin-resistant (RR)/multidrug-resistant TB (MDR-TB). Given the significant achievements in global treatment coverage, Indonesia needs to make special efforts to improve the screening and diagnosis of drug-resistant TB as well as access to treatment [1].

Rifampicin resistance has become a significant problem in TB control during long-term treatment with rifampicin. Resistance to this drug can lead to severe consequences, such as TB treatment failure, the prolongation of treatment, and increased retreatment rates. As very few mono-resistant strains are resistant to rifampicin, and most are also resistant to isoniazid, it has become a marker for MDR-TB [1, 4]. According to World Health Organization (WHO) guidelines, the anti-TB drugs used to treat RR/MDR-TB can be classified into groups A, B, and C. This new classification is based on the drug class and the certainty of the evidence about the drugs' efficacy and safety. The drugs in these groups play a therapeutic role in the MDR-TB regimen [5]. The efficacy of bedaquiline (group A) in patients with pulmonary MDR-TB was first demonstrated in 2014 when the addition of bedaquiline to the regimen showed a faster and increased number (78%) of culture conversions after 120 weeks compared with placebo (58%) [6]. MDR-TB patients treated with bedaquiline also had TB-negative sputum within six months [7, 8]. Bedaquiline is strongly recommended for the long-term treatment of patients with RR/MDR-TB [9]. Currently, bedaquiline resistance mechanisms include mutations in the *atpE* and cross-resistance to clofazimine in the *Rv0678*, *Rv1979c*, and *pepQ* genes, with linezolid resistance encoded by the *rrl* and *rplC* genes.

Bedaquiline inhibits adenosine triphosphate (ATP) synthesis and several resistance-associated mutations encoded by the *atpE* gene as an additional resistance mechanism [10, 11]. Mutations in the *atpE* are also associated with resistance both in vitro and in vivo [6, 10, 12, 13], and a high mutation frequency of *Rv0678* contributes to low resistance to bedaquiline in vitro and clinically. In 2016, the mutations in *Rv1979c* and *pepQ* were found to be associated with bedaquiline and clofazimine resistance. *Rv0678*, *Rv1979c*, and *pepQ* also have cross-resistance between bedaquiline and clofazimine [13–17].

The distribution of *M. tuberculosis* strains in various regions shows variations in the level of virulence in the process of human adaptation, with epidemiological differences dominating. Currently, *M. tuberculosis* has nine lineage strains distributed across many parts of the world [18]. The lineages include Indo-Oceanic (L1), East Asian/Beijing (L2), East African-Indian (L3), European-American (L4), West African 1 (L5), West African (L6), Aethiops vetus (L7) [19], and East African (L8 [20] and L9 [18, 21]). Several molecular identification methods have been developed from these strains of *M. tuberculosis*, including IS6110-RFLP, MIRU-VNTR, and spoligotyping. These methods have demonstrated high resolution and good performance in clinical trials, tracing, and reinfection detection. However, the great diversity or, in some cases, excessive homogeneity makes the application of this method unsuitable for the phylogenetic analysis of strains of *M. tuberculosis* [22, 23]. Whole-genome sequencing has facilitated advances in the study of *M. tuberculosis* resistance, transmission kinetics, and phylogenetic analysis, as well as developments in sequencing technology [23]. In this study, we aimed to determine the relationship between lineage and bedaquiline, clofazimine, and linezolid resistance based on the phenotypic profiles of *M. tuberculosis* RR isolates from West Java, Indonesia, using whole-genome sequencing technology.

## 2. Materials and Methods

### 2.1. Isolates Collection and Identification

This study was conducted at the Laboratory of the Department of Microbiology, Faculty of Medicine, Universitas Indonesia. A total of 60 isolates from the stock of the Department of Microbiology, Faculty of Medicine, Universitas Indonesia, were subcultured on a Löwenstein–Jensen (LJ) medium and identified as *M. tuberculosis* bacteria using the SD MPT64 Rapid test (SD Bioline) to ensure all the bacteria are *M. tuberculosis* and not contaminated by other bacteria. Previously before bacterial stock was made, we tested the phenotype of bacteria to rifampicin using the GeneXpert method and to bedaquiline, clofazimine, and linezolid using the mycobacteria growth indicator tube (MGIT) liquid culture method.

### 2.2. Genomic DNA Isolation and Whole-Genome Sequencing

Genomic DNA extraction from the *M. tuberculosis* isolates was conducted using the modified cetyl trimethyl ammonium bromide (CTAB) method. Briefly, 3-4 *M. tuberculosis* colonies from the LJ medium were scrapped and transferred into a screw cap tube containing 100 *μ*L Tween 80 and 6–8 sterile beads and then vortexed. Thereafter, 1 mL of nuclease-free water was added to each tube and vortexed until a bacterial suspension was obtained. A 300 *μ*L bacterial suspension was transferred to a microcentrifuge tube, heated in a water bath for 30 min at 95°C, and cooled to room temperature. Subsequently, 50 *μ*L lysozyme (10 mg/mL) was added to each tube, which was then vortexed and incubated for 2 hours at 37°C. Following incubation, a mixture of 70 *μ*L 10% SDS and 5 *μ*L proteinase *K* was added to each tube, which was then vortexed and incubated for 10 minutes at 65°C. Then, 100 *μ*L 5M NaCl and 100 *μ*L of CTAB-NaCl solution preheated to 65°C were added. The mixture was vortexed until it turned milky and then incubated for 10 min at 65°C. A 750 *μ*L of chloroform/isoamyl alcohol (24 : 1) solution was added, vortexed for 1 minute, and centrifuged at 12,500 rpm for 8 min at room temperature. Next, 200 *μ*L supernatant was transferred to a tube, and 300 *μ*L of cold isopropanol was added. This was mixed with the tube inverted 2–3 times and then incubated for 2 hours at −20°C. DNA pellets were obtained by centrifugation at 12,500 rpm for 15 min at 4°C. The supernatant was removed and added 1 mL cold ethanol 70%, and then inverted 2-3 times. DNA pellets were obtained by centrifugation at 12,500 rpm for 5 min at 4°C. The centrifugation was repeated at 12,500 rpm for 2 min at 4°C, and the remaining ethanol was removed with a micropipette and dried. Finally, 37 *μ*L of nuclease-free water was added. The quality and quantity of the genomic DNA were measured using a NanoDrop A260/280, Qubit Fluorometer, and agarose gel electrophoresis. The DNA samples were sent to Omics Drive Pte Ltd Co. for sequencing using a NovaSeq 6000 (Illumina). Library preparation used Illumina DNA Prep Kit and was undertaken according to the guidelines, and the library pools were subjected to single-end sequencing (150 bp).

### 2.3. Bioinformatics and Data Analysis

Omics Drive Pte Ltd Co. performed the following bioinformatics analysis. FastQC v0.11.9 was used to check the quality of the raw sequences. Trimmometric v0.39 was used to eliminate any poor-quality reads. The high-quality reads were mapped using BWA v0.7.17. The SAM format data were converted to BAM format, and the BAM files were sorted and indexed using SAMtools v1.15.1. SPAdes v3.15.3 was used for the de novo assembly. GATK-HaplotypeCaller was used for the variant calling and SnpEff v4.3 for the variant annotations. The analysis information is then compiled into a file. Mykrobe v0.10 and TB-Profiler v4.4.1 were used to predict the lineage classification, and only Mykrobe was used to predict the type of drug resistance among the isolates [24–27]. Descriptive statistics were used to describe the results of this study.

### 2.4. Ethics Approval of Research

The Health Research Ethics Committee of the Faculty of Medicine, Universitas Indonesia, Jakarta, Indonesia, approved the study protocol (protocol no. 22-12-1429) and issued it on December 5th, 2022.

## 3. Results and Discussion

### 3.1. *M. tuberculosis* Phenotypic DST Profile Characteristics

**Figure 1:**
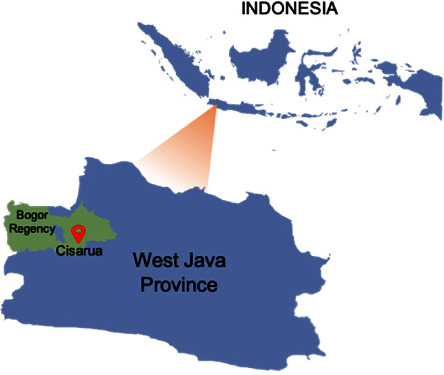
MTB RIF‐resistance samples origins in West Java, Indonesia.

The geographical origins of the studied strains from West Java are shown in Figure 1. In total, 397,377 cases of TB were recorded in 2021 with 1.5% of them are rifampicin-resistant (RR)/MDR-TB; West Java province reported the most significant number of cases at 91,368 with a detection rate of 90.6%, and the data thus accurately depicted the distribution of *M. tuberculosis* strains in this population. Many people still have pulmonary TB, and the number of new cases being identified each year is increasing due to a lack of public awareness of the available TB treatments [28, 29].

Sixty *M. tuberculosis* RR isolates from Cisarua, West Java, were available for drug susceptibility testing with bedaquiline, clofazimine, and linezolid. We found that while 5% of the isolates were resistant to bedaquiline, 100% were phenotypically sensitive to linezolid and clofazimine (Table 1). Bedaquiline is a novel regimen that is beneficial for treating TB, as the administration of this drug can convert TB-positive sputum to negative within six months [7, 8]. Bedaquiline is used to treat MDR-TB patients via the BPal (bedaquiline, pretomanid, and linezolid) regime in some provinces in Indonesia. The data from our study indicate that resistance to bedaquiline has emerged. Although minimal, it serves as a warning, and a strategy to prevent bedaquiline resistance from increasing must be prepared.

In 2005, the U.S. Centers for Disease Control and Prevention introduced the concept of TB infected by *M. tuberculosis* extremely drug-resistant (XDR) as a different entity. These bacteria cannot be killed by fluoroquinolone therapy, such as levofloxacin and moxifloxacin, or other second-line drugs, such as amikacin and kanamycin, and are resistant to first-line drugs such as isoniazid and rifampicin. XDR *M. tuberculosis* can occur due to the inappropriate handling of MDR-TB patients [20, 30]. On the other hand, phenotypic studies of bedaquiline, clofazimine, and linezolid to RR background (RR, MDR, and XDR) *M. tuberculosis* isolates were low. However, the data indicated that coresistance to bedaquiline, linezolid, and clofazimine was still very low in the tested population [31]. Recently, a series of clinical trials evaluating new and alternative oral anti-TB drugs (bedaquiline, clofazimine, and linezolid) have demonstrated their potential in treating resistant TB in a shorter time with lower toxicity and higher efficacy [32]. It was previously believed that phenotypic drug susceptibility testing (DST) employing automated methods like the MGIT 960 system could accurately detect *M. tuberculosis* susceptibility or resistance to first-line anti-TB medications [33]. However, these techniques require considerable time and effort and produce results in weeks rather than days [33, 34]. Methods that address these limitations are thus needed as alternatives or companions to culture-based methods. Whole-genome sequencing has emerged as an alternative to further understand resistance profiles and lineage dissemination within a country [23, 33]. This method has been implemented in many countries, primarily to detect the resistance profiles of first-line anti-TB drugs and to conduct epidemiological studies [4, 18]. We undertook whole-genome sequencing using genes possibly associated with *M. tuberculosis* resistance to bedaquiline and identified mutations linked to these phenotypic traits. These findings demonstrate that the whole-genome sequencing method can serve as a suitable alternative alongside culture methods or potentially replace them.

### 3.2. Whole-Genome Sequencing Results of *M. tuberculosis* Isolates

Our tools for genome mutation analysis, Mykrobe and TB-Profiller, have long been widely used. These two tools promise to make it easy for users to analyze antituberculosis drug resistance profiles and determine lineage quickly [35, 36].

**Figure 2:**
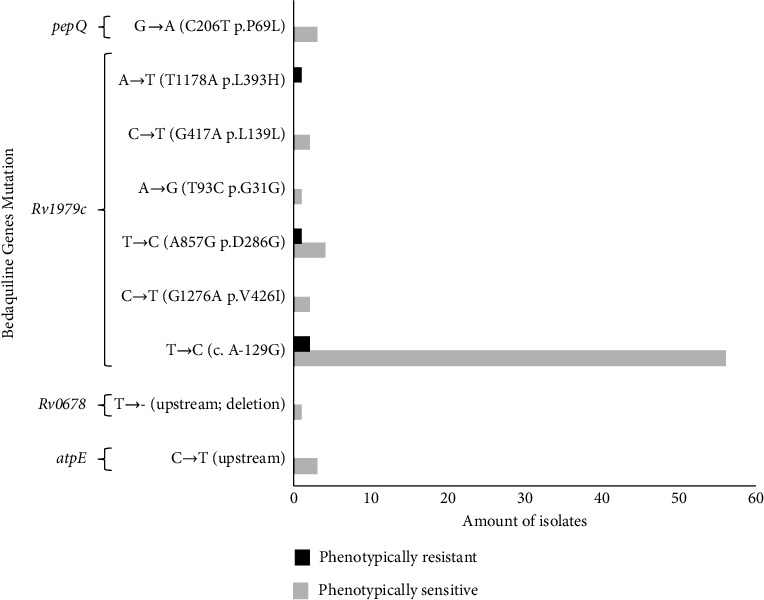
The distribution of mutations encoding bedaquiline resistance genes was discovered using a whole‐genome sequencing study of 60 *M. tuberculosis* isolates.

The genes encoding bedaquiline resistance, specifically *atpE*, *Rv0678*, *Rv1979c*, and *pepQ*, was then looked for in the data collected from the WGS study.  Figure 2 displays the distribution of bedaquiline-encoding gene variants discovered using WGS. Out of the 60 isolates, three isolates were found to be phenotypically resistant to bedaquiline; however, only two isolates were found to have gene mutations, and ten isolates were found to be phenotypically sensitive but harbored mutations in the bedaquiline resistance gene. The *Rv1979c* T⟶C (c. A-129G) gene, located upstream of the *Rv1979c* gene, was determined to be the most often detected mutation in all isolates according to the findings of this investigation. One of the 2/60 phenotypically and genotypically resistant organisms has the *Rv1979c* gene mutation, which is linked to bedaquiline resistance in Asp286Gly and Leu393His. Val426Ile, Asp286Gly, Gly31Gly, and Leu139Leu were among the other variants in the *Rv1979c* gene that were discovered, but they were shown to be phenotypically sensitive. In the upstream region of the *atpE* gene, 3/60 mutations (C⟶T) were discovered, but in the upstream region of the *Rv0678* gene, only 1/60 isolates (T⟶−) were discovered. The *pepQ* G⟶A (Pro69Leu) gene mutation was discovered in the genotypically but phenotypically sensitive strains (3/60).

Numerous studies have reported various mutations discovered as a result of WGS analysis. Rv1979c and *pepQ* gene alterations were reported by Ismail et al. [15]. According to the research by Ismail et al. [15], the Rv1979c gene missense mutation Asp286Gly and the synonymous mutations Leu139Leu and Gly31Gly were discovered in *M. tuberculosis* isolates that had drug susceptibility test findings that showed they were sensitive to bedaquiline phenotypically. The WHO database of resistance coding gene variants has confirmed the mutation often discovered in this study, which is the mutation at codon position −129 (A⟶G) in the upstream region of the Rv1979c gene, and it does not correlate with bedaquiline resistance [37].

Both of those genes, the efflux pump repressor *mmpR* (*Rv0678*) and the *atpE* gene (*Rv1305*), specifically the F0 domain of the ATP synthase enzyme, exhibit alterations in bedaquiline-producing mutants that are resistant to the drug [38, 39]. The uncharacterized transporter *Rv1979c* and *pepQ*, which are both encoded as genes involved in cross-resistance between bedaquiline and clofazimine, have statistically significant co-occurrences with *Rv0678* [40, 41]. However, the connection between bedaquiline and clofazimine resistance in these two genes (*Rv1979c* and *pepQ*) has to be explored further. Mutations in the *M. tuberculosis* genome can alter drug targets or metabolic pathways involved in drug resistance. These mutations can have a significant impact on the effectiveness of antituberculosis drugs [42]. Drug resistance in *M. tuberculosis* is a complicated phenomenon with numerous interrelated genetic components. Drug resistance can result from changes in a single gene or a combination of abnormalities in several genes connected to the resistance pathway [43].

### 3.3. *M. tuberculosis* Resistance Type and Lineage Distribution

We found agreement between the drug-resistant type of RR/MDR *M. tuberculosis* from the genotypic results using Mykrobe analysis and the phenotypic DST results for bedaquiline (Table 2). The studied strains from the genotypic results showed 88.33% (53/60) were RR *M. tuberculosis*, and the remainder had an MDR 11.67% (7/60) background (Table 2). Table 2 demonstrates the association between anti-TB drug resistance to rifampicin (RR or MDR) and bedaquiline. Our results revealed that one bedaquiline-resistant isolate had an RR background and two had an MDR background. This discovery is crucial as it demonstrates the occurrence of bedaquiline-resistant cases within the context of RR/MDR *M. tuberculosis*. The WHO recommends bedaquiline as one of the three essential medications that must be used to treat RR-TB in every regimen.

Notwithstanding, bedaquiline resistance was already noted shortly following its introduction. The establishment of bedaquiline resistance and difficulties in measuring resistance to bedaquiline make it difficult to employ bedaquiline effectively [44]. MDR-TB and RR-TB are both conditions that can be treated with bedaquiline [45]. According to a recent cost-effectiveness study, adding bedaquiline to a baseline MDR-TB regimen will enhance health outcomes and lower costs in high TB environments [46]. With success rates as high as 78% and fewer fatalities compared to normal regimens, bedaquiline has been proven to be successful in treating MDR-TB [46, 47].

Several factors can affect discrepancies between phenotypic and genotypic data. According to numerous studies, quiet or disputed mutations and heteroresistance are the most frequent reasons for discrepancies between phenotypic and genotypic techniques when testing drug resistance in TB. Silent mutations in *M. tuberculosis* DNA affect the nucleotide but not the amino acid. Some disputed variants in the gene are found genotypically but produce drug-resistance results in phenotypic testing, particularly in liquid media (MGIT 960). These mutations affect the “fitness” of *M. tuberculosis*, causing it to evolve slowly in the presence of the drugs during phenotypic DST, which prevents it from being detected [48].

In this study, 48.33% of the isolates belonged to the Euro-American lineage (L4), with most of them being of the Haarlem clade, followed by the East Asia/Beijing lineage (L2) at 46.67% and the Indo-Oceanic lineage (L1) at 5% (Table 3).

**Figure 3:**
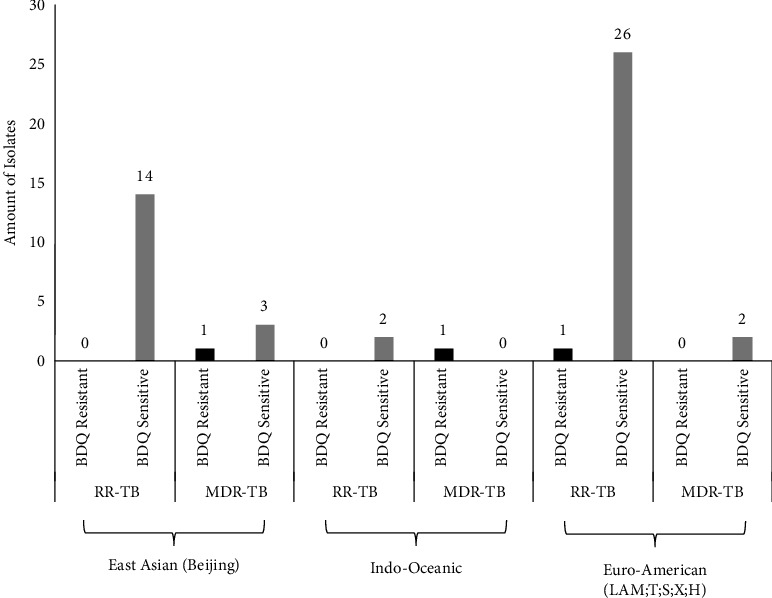
MTB lineage distribution and drug resistance type.

Lineage 4 has also been found in Upper Myanmar, which could be explained by cross-border transmission with Thailand. Information about the future distribution of these *M. tuberculosis* strains can be linked to information across borders to assess transboundary transmission [49, 50]. In a recent TB study from Thailand [51], L2 was implicated in the increased incidence of MDR-TB, as it is associated with drug resistance and increased transmissibility. Another possibility could be that these isolates were part of an outbreak, but as this is an archived collection with no epidemiological data, we could not investigate further. In Cambodia, the majority of the lineages are also L1 (Indo-Oceanic), L2 (East Asian), and L4 (Euro-American) [52]. The high prevalence of the L1 *M. tuberculosis* strain in Cambodia is perhaps not surprising as it is a common strain in the Southeast Asian countries of India and Bangladesh [51]. The distribution between lineage and drug resistance type is shown in Figure 3.

In this study, we aimed to establish knowledge regarding the type distribution of *M. tuberculosis* RR isolates in Indonesia, especially in West Java province. Such a knowledge base is essential for epidemiological research aimed at infection control and in helping to provide effective antibiotic therapy. Furthermore, detecting drug-resistant TB in Indonesia is reliant on the use of Xpert *M. tuberculosis*/rifampicin and line probe assays (MTBDRplus and MTBDRsl), which are limited to detecting resistance in only first- and second-line drugs and not the new anti-TB drugs used to treat MDR-TB [24]. Next-generation sequencing (NGS) technology, especially for TB, provides the most comprehensive approach to molecular-based DST. Whole-genome sequencing is a method of determining the exact nucleotide sequence of a particular genome, which is the entire genetic material of an organism [53, 54]. NGS can sequence millions to billions of reads in a single process within a shorter time and with more accurate results, which makes it a cost-effective and powerful sequencing technology with high-throughput data [55, 56]. The results concordance data between Xpert MTB/RIF, a line probe assay, and whole-genome sequencing with phenotypic DST to predict resistance to 15 anti-TB drugs were reported as 40%, 63%, and 93%, respectively [57].

The results of this study are fascinating but also limited to the conditions of the origin of the samples. In addition, this study only focuses on in vitro phenotypic profiles and does not involve the clinical status of patients. To fully understand the role of specific mutations in conferring resistance to TB drugs, further data on WGS and phenotypic testing are required, especially for isolates that are resistant to bedaquiline, clofazimine, and linezolid.

## 4. Conclusions

This study demonstrates the applicability and value of WGS in identifying drug resistance and estimating disease transmission in West Java. Some mutations were found to support phenotypically resistant. However, this study revealed an uncorrelation between specific lineage and phenotype profiles where the bedaquiline resistance was found in all lineages. All the mutation data will be a helpful database to determine antituberculosis drug choice if the WGS is used for a laboratory tool examination in Indonesia. Our study further showed that the *M. tuberculosis* isolates from West Java, Indonesia, used in this study predominantly belonged to the Euro-American lineage, followed by the East Asian and Indo-Oceanic lineages.

## Figures and Tables

**Table 1:** Phenotypic DST results for anti-TB drugs.

Anti-TB drugs	Amount of isolates *n*	Resistant *n* (%)	Sensitive *n* (%)
Bedaquiline	60	3 (5)	57 (95)
Clofazimine	60	0 (0)	60 (100)
Linezolid	40	0 (0)	40 (100)

**Table 2:** MTB isolates characteristics based on Mykrobe analysis.

Drug resistance type	Bedaquiline status	Number (%)	Total
RR	Resistance	1 (1.89)	53
	Sensitive	52 (98.11)	

MDR	Resistance	2 (28.57)	7
	Sensitive	5 (71.42)	

Total			60

**Table 3:** Lineage distribution based on TB-Profiler analysis.

Lineage distribution	Number	Percentage (%)
Indo-Oceanic (L1)	3	5
East Asian/Beijing (L2)	28	46.67
Euro-American (L4)	29	48.33

## Data Availability

The data used to support the findings of this study are available from the corresponding author upon reasonable request.
